# Supportive Management of Severe Acetaminophen and Ibuprofen Overdose Twenty-Four Hours After Ingestion in Limited Resources Settings

**DOI:** 10.14740/jmc5330

**Published:** 2026-06-03

**Authors:** Alert Drishti, Entela Shkodrani, Rudin Domi, Ergita Nelaj, Gentian Huti, Asead Abdyli, Filadelfo Coniglione, Krenar Lilaj, Majlinda Naco, Alma Cani, Vedat Eljezi

**Affiliations:** a Department of Surgery, Service of Clinic Toxicology, University of Medicine, Tirana, Albania; b Department of Infectious Diseases, Service of Dermatology, University of Medicine, Tirana, Albania; c Department of Surgery, Service of Anesthesia and Intensive Care, University of Medicine, Tirana, Albania; d Department of Internal Medicine, Service of Internal Medicine, University of Medicine, Tirana, Albania; e Department of Anesthesiology and Intensive Care Medicine, American Hospital 3, Tirana, Albania; f Department of Perioperative Medicine, CHU Gabriel-Montpied, Clermont-Ferrand, France

**Keywords:** Toxicology, Overdose, Acetaminophen, Ibuprofen, Supportive treatment, Intensive care unit

## Abstract

Acetaminophen and ibuprofen are among the most used analgesic–antipyretic agents worldwide, yet their combined overdose can become life-threatening, particularly when medical care is delayed. We report the case of a 29-year-old woman who presented to the emergency department more than 48 h after ingesting a massive dual overdose of acetaminophen (25 g) and ibuprofen (20 g). At admission, she complained of nausea, epigastric pain, tinnitus, and mild jaundice. Laboratory findings indicated severe acute liver injury, with markedly elevated transaminases, hyperbilirubinemia, significant coagulopathy, and metabolic acidosis, consistent with advanced acetaminophen toxicity. Due to the delayed presentation, the Rumack–Matthew nomogram was no longer reliable for risk assessment. Although antidotal therapy was indicated, neither N-acetylcysteine nor alternative antidotes were available, and options such as hemodialysis or liver transplantation were inaccessible. Management therefore relied entirely on intensive supportive care, including correction of metabolic disturbances, coagulopathy, and evolving organ dysfunction, alongside close clinical and biochemical monitoring. Despite the severity of hepatic failure and metabolic derangement at presentation, the patient showed gradual improvement over a prolonged hospital course. After 53 days of supportive therapy, liver function recovered sufficiently, and transplantation was not required according to King’s College criteria. This case illustrates that even in severe, late-presenting combined analgesic overdose complicated by acute liver failure and metabolic acidosis, a favorable outcome may still be achieved with comprehensive supportive care alone when advanced therapies are unavailable. It underscores the pivotal role of meticulous supportive management in toxicological emergencies.

## Introduction

Acetaminophen and ibuprofen are among the most widely used analgesic–antipyretic agents globally. While their combined ingestion does not appear to enhance the intrinsic toxicity of either drug, overdose significantly increases the risk of serious systemic complications, as demonstrated in our case. Paracetamol poisoning remains one of the most frequent causes of drug overdose worldwide, accounting for approximately 6% of all poisoning cases and up to 56% of severe acute liver injury and acute liver failure in reported regions [1]. Despite its high incidence, outcomes are generally favorable when treatment is initiated early, with an estimated case fatality rate of 0.4% under modern management, largely attributable to the effectiveness of N-acetylcysteine (NAC) [2]. However, delayed presentation such as in our patient, substantially increases the likelihood of severe hepatotoxicity, progression to acute liver failure, and death. Within this context, our case emphasizes the importance of timely and optimized supportive therapy, especially in resource-limited settings where advanced liver support systems such as molecular adsorbent recirculating system (MARS) and specific antidotes (e.g., methionine or fomepizole) may not be available. Under these constraints, meticulous intensive care, encompassing hemodynamic stabilization, correction of metabolic disturbances, renal support, and prevention of complications, become the cornerstone of management and may be lifesaving. Thus, although overall mortality remains relatively low, the high prevalence of exposure and its role as a leading cause of acute liver failure make paracetamol toxicity a major public health concern. In our setting, at the Clinical Toxicology Service of the “Mother Teresa” University Hospital Center, combined intoxications are frequently encountered and represent true medical emergencies. Delayed hospital presentation, as seen in this case, further worsens outcomes and limits available therapeutic options.

## Case Report

### Investigations

A 29-year-old non-pregnant woman was admitted to the emergency department with nausea, abdominal pain, and fever. She had no relevant past medical history, was using oral contraceptives, and denied recreational drug use. On admission, vital signs were within normal limits. Physical examination revealed mild scleral icterus. In the absence of an initial toxicological history, standard supportive management was initiated while diagnostic laboratory investigations were underway.

### Diagnosis

The occurrence of tinnitus and jaundice, together with laboratory findings of total bilirubin 3.04 mg/dL (normal range < 1.2 mg/dL), alanine aminotransferase (ALT) 726 U/L (normal range < 35 U/L), aspartate aminotransferase (AST) 728 U/L (normal range < 43 U/L^)^, prothrombin time (PT) 23%, international normalized ratio (INR) 3.29 (normal range 0.8–1.2), arterial pH 7.333, partial pressure of carbon dioxide (PaCO_2_) 34.5 mm Hg, bicarbonate (HCO_3_^−^) 17.9 mm Hg, and base excess −7.0 mmol/L, raised strong suspicion of acute drug-induced liver injury (DILI) secondary to acetaminophen and non-steroidal anti-inflammatory drug (NSAID) overdose. This was confirmed 1 h and 25 min after emergency department admission, when the patient disclosed a deliberate suicidal self-poisoning with 25 g of immediate-release acetaminophen and 20 g of ibuprofen, reportedly occurring more than 48 h before hospital admission. The timing was based on the patient’s report and could not be precisely established; however, the laboratory findings were consistent with an ingestion occurring approximately more than 48 h before presentation.

### Treatment

The presence of jaundice together with markedly elevated serum transaminases indicated progression to the hepatotoxic phase (phase II–III, > 24 h post-ingestion) of acetaminophen-induced acute liver injury, with evolving acute liver failure and its systemic consequences. At this stage, the Rumack–Matthew nomogram is no longer reliable for risk stratification; however, current toxicology guidelines strongly recommend immediate initiation of antidotal therapy, as delayed treatment may still provide hepatoprotective benefit and limit progression to fulminant hepatic failure. In this case, due to the unavailability of methionine and fomepizole, specific antidotal therapy could not be administered, and management was therefore restricted to intensive supportive care. Even out of time limits, NAC was administered. The clinical course was complicated by hepatic, renal, and hemodynamic dysfunction, consistent with multi-organ involvement secondary to acute liver failure. Renal replacement therapy is generally indicated not only for metabolic acidosis, electrolyte disturbances (notably hyperkalemia), and severely reduced glomerular filtration rate (GFR < 15 mL/min), but also for uremic manifestations, including profound fatigue, altered mental status, and seizures, as well as for its potential contribution to toxin and metabolite clearance. However, in our patient, the indications for renal replacement therapy were not reached, therefore it was not initiated. Advanced extracorporeal liver support systems, such as single-pass albumin dialysis (SPAD) and the MARS, which may enhance hepatic detoxification and reduce circulating bilirubin, ammonia, and lactate levels, were not available in our setting. Consequently, referral to a specialized center for urgent liver transplantation in the context of acute liver failure was initiated. Supportive management in the intensive care unit was directed toward stabilization of hepatic function, renal impairment, and hemodynamic status, while preventing complications of liver failure, including coagulopathy, encephalopathy, hypoglycemia, and thermoregulatory instability. Oxygen therapy was administered via face mask at 3–5 L/min, with continuous capnography and serial arterial blood gas monitoring. Hemodynamic support included cautious volume resuscitation with Ringer’s lactate, recognizing its potential hypotonicity and the need to avoid exacerbation of intracranial pressure in the setting of hepatic encephalopathy. Metabolic acidosis was corrected with intravenous sodium bicarbonate (8.4% diluted in 5% glucose), with dosing guided by the standard formula: HCO_3_^−^ deficit (mEq) = 0.5 × body weight (61 kg) × (target HCO_3_^−^ (24 mEq/L) − measured HCO_3_^−^). Initially, 50% of the calculated deficit was administered, followed by reassessment and titration based on repeat arterial blood gas analysis. Electrolyte imbalances were carefully corrected using intravenous potassium chloride, calcium chloride, and magnesium sulfate with close laboratory monitoring guiding therapy. Strict temperature monitoring was maintained using a continuous skin probe, and hypothermia was actively prevented through warming blankets and warmed intravenous fluids, given its association with worsened coagulopathy and hemodynamic instability in liver failure. Hypoglycemia, a frequent consequence of impaired hepatic gluconeogenesis, was treated with 40% glucose and continuous monitoring. Coagulopathy, reflecting reduced hepatic synthesis of clotting factors, was managed with platelet transfusions, fresh frozen plasma, and vitamin K, guided by PT, INR, and clinical evidence of bleeding. Hepatic encephalopathy was addressed with lactulose to reduce ammonia absorption and production, along with gastrointestinal decontamination using neomycin and activated charcoal. Additional hepatoprotective measures included administration of vitamin E and silymarin, while ursodeoxycholic acid was used to support bile flow and manage hyperbilirubinemia. Overall, management focused on mitigating the systemic consequences of acute liver failure, including metabolic derangements, renal dysfunction, circulatory instability, and neurological complications, while arranging definitive treatment through liver transplantation.

### Follow-up and outcome

**Figure 1:**
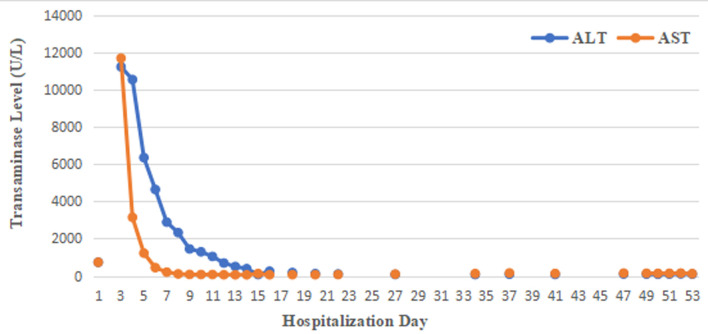
Transaminase levels over time during hospitalization.

**Figure 2:**
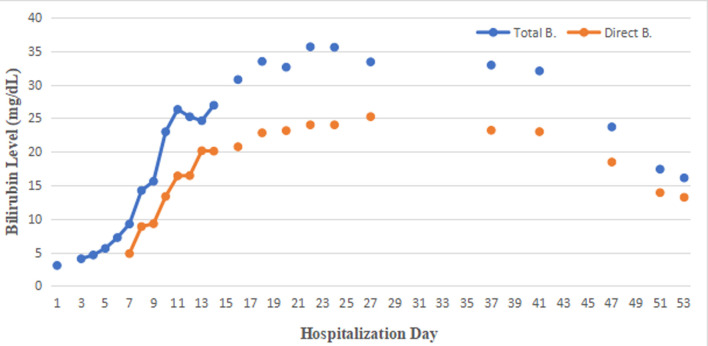
Blood bilirubin levels over time during hospitalization.

**Figure 3:**
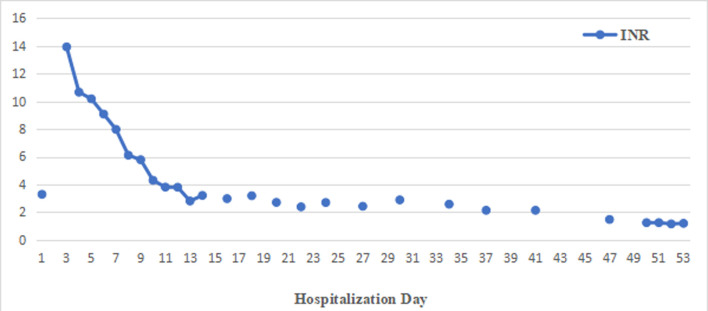
The international normalized ratio (INR) over time during hospitalization.

**Figure 4:**
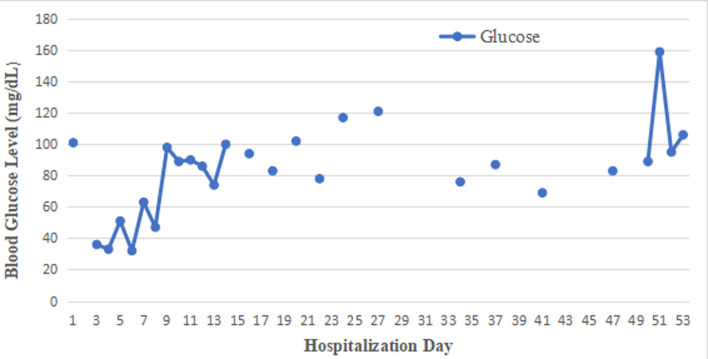
Blood glucose levels over time during hospitalization.

**Figure 5:**
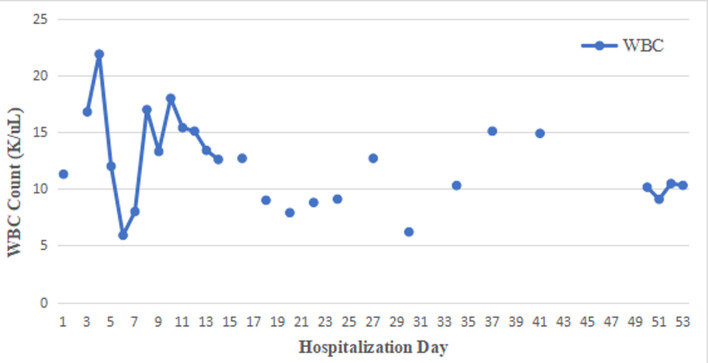
White blood cell (WBC) count over time during hospitalization.

**Figure 6:**
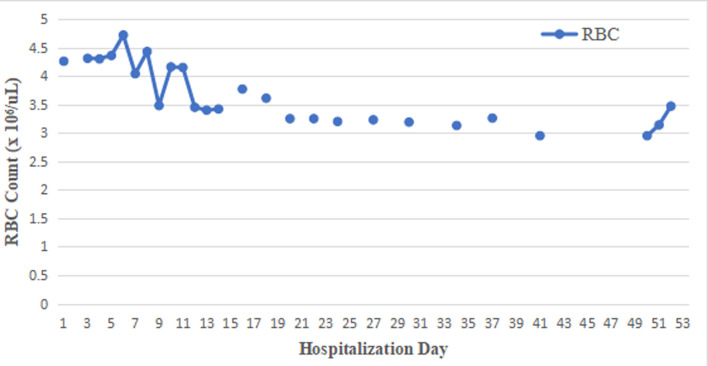
Red blood cell (RBC) count over time during hospitalization.

**Figure 7:**
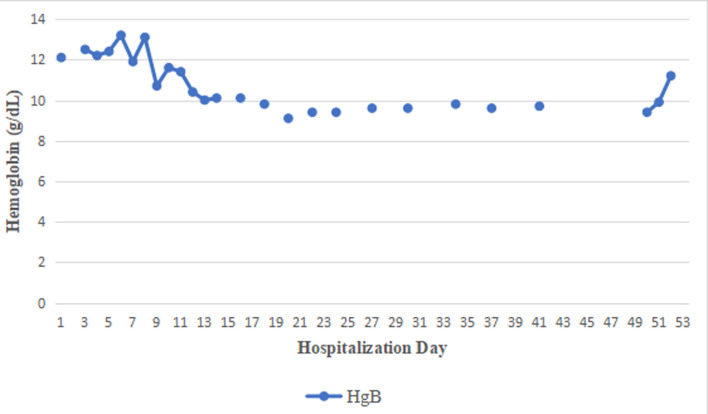
Hemoglobin levels over time during hospitalization.

**Figure 8:**
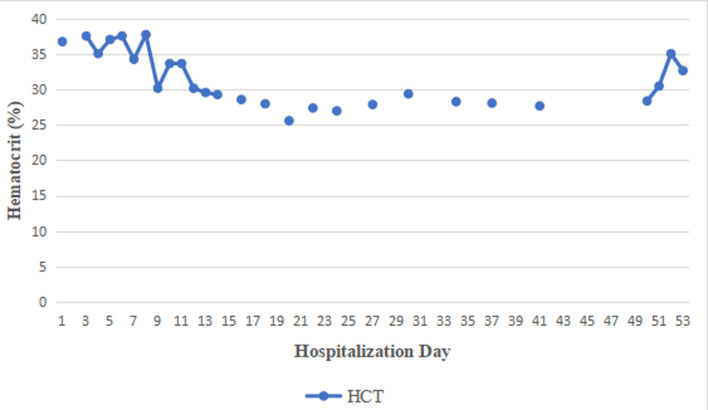
Hematocrit levels over time during hospitalization.

**Figure 9:**
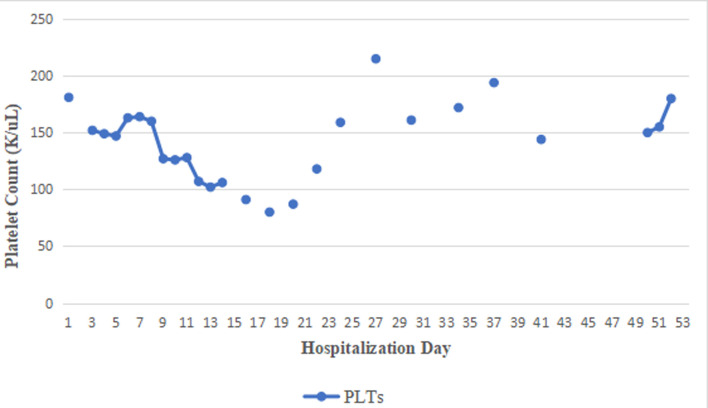
Platelet count over time during hospitalization.

Marked laboratory derangements were observed during the clinical course, with a peak AST of 11,688 U/L and ALT of 11,224 U/L, both recorded at day 3, accompanied by a profoundly elevated INR of 13.91 on the same day, reflecting severe hepatic injury and coagulopathy. Total bilirubin reached its maximum value of 35.65 mg/dL on day 22, indicating prolonged cholestasis. A nadir glycemia of 32 mg/dL was documented on day 6, consistent with significant metabolic impairment. Additional findings included marked leukocytosis, with white blood cell counts rising to 21,900 k/µL, progressive anemia with hemoglobin levels around 9–10 g/dL, and thrombocytopenia ranging between approximately 80,000 and 100,000 k/µL. Mild electrolyte disturbances were noted throughout the course, while renal function remained preserved. Notably, all microbiological cultures were negative, arguing against a superimposed infectious process. Despite these severe biochemical abnormalities, she showed progressive improvement with supportive care, including normalization of liver enzymes, improvement in coagulation parameters, and a gradual decline in bilirubin levels. The patient ultimately achieved complete clinical recovery. Tables 1–9 and Figures 1–9 illustrate the trends in transaminases, bilirubin, INR, glycemia, white and red blood cell counts, hemoglobin and hematocrit, and platelet count throughout hospital treatment.

**Table 1:** Transaminase Levels Over Time During Hospitalization

	Day													
	1	3	4	5	6	7	8	9	10	11	12	13	14	16
ALT	726	11,224	10,540	6,349	4,637	2,881	2,319	1,442	1,288	1,039	686	498	383	247
AST	728	11,688	3,139	1,217	438	197	101	73	69	69	57	54	52	56
	18	20	22	24	27	34	37	41	47	49	50	51	52	53
ALT	174	115	95	88	70	66	84	72	95	90	82	91	104	88
AST	60	59	67	79	90	120	153	125	144	138	132	135	145	115

ALT: alanine aminotransferase; AST: aspartate aminotransferase.

**Table 2:** Blood Bilirubin Levels Over Time During Hospitalization

	Day											
	1	3	4	5	6	7	8	9	10	11	12	13
Total	3.04	4.04	4.60	5.58	7.19	9.22	14.22	15.59	22.98	26.31	25.22	24.63
Direct						4.81	8.84	9.27	13.33	16.40	16.44	20.15
	14	16	18	20	22	24	27	37	41	47	51	53
Total	26.93	30.76	33.47	32.61	35.65	35.58	33.40	32.92	32.05	23.7	17.4	16.1
Direct	20.09	20.74	22.82	23.15	24.01	24.43	25.23	23.20	22.99	18.46	13.90	13.20

**Table 3:** The INR Over Time During Hospitalization

	Day													
	1	3	4	5	6	7	8	9	10	11	12	13	14	16
INR	3.29	13.91	10.65	10.17	9.07	7.97	6.11	5.78	4.30	3.81	3.80	2.81	3.21	2.98
	18	20	22	24	27	30	34	37	41	47	50	51	52	53
INR	3.19	2.71	2.39	2.70	2.43	2.89	2.58	2.14	2.14	1.48	1.25	1.25	1.16	1.20

INR: international normalized ratio.

**Table 4:** Blood Glucose Levels Over Time During Hospitalization

	Day													
	1	3	4	5	6	7	8	9	10	11	12	13	14	16
Glucose	101	36	33	51	32	63	47	98	89	90	86	74	100	94
	18	20	22	24	27	34	37	41	47	50	51	52	53	
Glucose	83	102	78	117	121	76	87	69	83	89	159	95	106	

**Table 5:** WBC Count Over Time During Hospitalization

	Day													
	1	3	4	5	6	7	8	9	10	11	12	13	14	16
WBC	11.3	16.8	21.9	12.0	5.9	8.0	17.0	13.3	18.0	15.4	15.1	13.4	12.6	12.7
	18	20	22	24	27	30	34	37	41	50	51	52	53	
WBC	9.0	7.9	8.8	9.1	12.7	6.2	10.3	15.1	14.9	10.15	9.08	10.47	10.31	

WBC: white blood cell.

**Table 6:** RBC Count Over Time During Hospitalization

	Day													
	1	3	4	5	6	7	8	9	10	11	12	13	14	16
RBC	4.26	4.31	4.30	4.36	4.72	4.07	4.43	3.48	4.16	4.15	3.45	3.40	3.42	3.77
	18	20	22	24	27	30	34	37	41	50	51	52	53	
RBC	3.61	3.25	3.25	3.20	3.23	3.19	3.13	3.26	2.95	2.95	3.14	3.47	3.33	

RBC: red blood cell.

**Table 7:** HgB Levels Over Time During Hospitalization

	Day													
	1	3	4	5	6	7	8	9	10	11	12	13	14	16
HgB	12.1	12.5	12.2	12.4	13.2	11.9	13.1	10.7	11.6	11.4	10.4	10.0	10.1	10.1
	18	20	22	24	27	30	34	37	41	50	51	52	53	
HgB	9.8	9.1	9.4	9.4	9.6	9.6	9.8	9.6	9.7	9.4	9.9	11.2	10.9	

HgB: hemoglobin.

**Table 8:** HCT Levels Over Time During Hospitalization

	Day													
	1	3	4	5	6	7	8	9	10	11	12	13	14	16
HCT	36.8	37.6	35.1	37.1	37.6	34.3	37.8	30.2	33.7	33.7	30.2	29.6	29.3	28.6
	18	20	22	24	27	30	34	37	41	50	51	52	53	
HCT	28.0	25.6	27.4	27.0	27.9	29.4	28.3	28.1	27.7	28.4	30.5	35.1	32.7	

HCT: hematocrit.

**Table 9:** PLT Count Over Time During Hospitalization

	Day													
	1	3	4	5	6	7	8	9	10	11	12	13	14	16
PLTs	181	152	149	147	163	164	160	127	126	128	107	102	106	91
	18	20	22	24	27	30	34	37	41	50	51	52	53	
PLTs	80	87	118	159	215	161	172	194	144	150	155	180	177	

PLT: platelet.

## Discussion

In population-based studies, the incidence of acute overdose is estimated at approximately 21 cases per 100,000 persons per year, with around 5 per 100,000 requiring hospitalizations [3]. The burden remains considerable in high-income settings; in the United States alone, paracetamol toxicity accounts for nearly 56,000 emergency department visits and 2,600 hospitalizations each year [4]. In absolute numbers, it is responsible for roughly 500 deaths annually in the United States and 150–200 deaths per year in England and Wales, and it continues to represent a leading indication for liver transplantation worldwide [4]. Table 10 summarizes the four stages of acetaminophen-induced liver injury [5, 6]. The Service of Clinical Toxicology at the University Hospital Center “Mother Teresa,” Albania, admitted 46 patients with acetaminophen overdose and 19 with ibuprofen overdose in 2023, out of a total of 206 patients treated in the service. Elgingihy et al evaluated repeated exposure to paracetamol and ibuprofen in male albino mice, demonstrating hematological, histopathological, and molecular alterations with both agents. Ibuprofen was associated with more pronounced changes in blood indices, while each drug produced mild, dose-dependent structural alterations in organs such as the liver and kidneys without overt organ failure. At the molecular level, downregulation of tumor necrosis factor-α (TNF-α) and connexin 43 suggested early systemic effects. These findings indicate that even sub-chronic use at therapeutic levels may induce early biological changes, highlighting the need for cautious use and further long-term investigation [7]. Recent reports have described several antidotal strategies aimed at mitigating DILI, as reflected in European Association for the Study of the Liver (EASL) guidelines [8]. In DILI management, prompt discontinuation of the offending agent remains the primary intervention, as many cases improve spontaneously thereafter. The role of antidotes is limited: NAC is the principal evidence-based therapy, primarily indicated for acetaminophen hepatotoxicity and occasionally used in acute liver failure of other causes. For most drugs, specific antidotes are either unavailable or lack sufficient supporting evidence. Corticosteroids may be considered in selected immune-mediated cases, whereas ursodeoxycholic acid is not recommended routinely. When progression to acute liver failure occurs, management prioritizes supportive care and liver transplant evaluation, underscoring that, aside from NAC, treatment is largely supportive in nature [8]. Gartner et al investigated the clinical impact of fomepizole as an adjunct to standard NAC therapy in high-risk acetaminophen overdose. The addition of fomepizole, an inhibitor of cytochrome P450 2E1 (CYP2E1), was associated with improved outcomes, including reduced progression to severe hepatotoxicity and acute liver failure. Patients receiving combination therapy demonstrated more favorable trends in liver enzyme evolution and overall clinical course compared with standard treatment alone. These results suggest that fomepizole may limit toxic metabolite formation and provide benefit in selected high-risk cases, although further prospective data are required to define its role [9]. Nogue-Xarau et al reviewed five decades of clinical experience with NAC, emphasizing its transformative effect on the prognosis of acetaminophen poisoning. NAC replenishes glutathione stores, enhances detoxification of the toxic metabolite N-acetyl-4-benzoquinone imine (NAPQI), and exerts antioxidant and anti-inflammatory actions. Early administration markedly reduces hepatotoxicity and mortality, establishing NAC as the cornerstone of treatment. Over time, dosing regimens and routes of administration have been refined to improve safety and tolerability. Nonetheless, challenges persist in late-presenting cases and in optimizing treatment for high-risk patients, highlighting the need for ongoing refinement of therapeutic strategies [10]. Nakatsu et al conducted a systematic review and meta-analysis comparing two-bag versus traditional three-bag intravenous acetylcysteine regimens in paracetamol poisoning. Their findings show that the two-bag regimen achieves comparable efficacy in preventing hepatotoxicity while significantly reducing adverse reactions, particularly anaphylactoid events, and simplifying administration. It was also associated with fewer interruptions and medication errors. Overall, the study supports the two-bag protocol as a safe and effective alternative with practical advantages in clinical settings [11]. Muriel et al described methionine as a hepatoprotective agent owing to its central role in hepatic metabolism and antioxidant defense. As a precursor of S-adenosylmethionine (SAMe) and glutathione, it contributes to reducing oxidative stress, enhancing detoxification, and maintaining redox balance. Methionine also supports lipid metabolism, helping to limit hepatic steatosis, and exerts anti-inflammatory and antifibrotic effects through modulation of cytokine production and inhibition of stellate cell activation. Overall, it represents a potentially valuable therapeutic strategy in liver disease through combined metabolic and regulatory effects [12]. The Rumack–Matthew nomogram is a widely used clinical tool for assessing the risk of hepatotoxicity after acute acetaminophen overdose and for guiding the initiation of NAC therapy [13]. However, its applicability is limited to single acute ingestions with a clearly known time of exposure, and it is considered valid only when serum paracetamol concentrations are measured between 4 and 24 h after ingestion [14]. In our case, the patient presented more than 48 h after the reported ingestion, rendering the Rumack–Matthew nomogram clinically unhelpful. Nevertheless, NAC therapy was administered despite the delayed presentation because of the strong clinical and laboratory evidence of significant acetaminophen toxicity. Even when initiated beyond the conventional early window, antidotal therapy may still confer hepatoprotective effects, attenuate ongoing injury, and reduce progression to fulminant hepatic failure. This approach highlights that, despite limitations in available antidotes for DILI, NAC may remain beneficial even in late-presenting cases [15]. MARS is an advanced liver support modality used in severe liver failure, including DILI, when conventional therapy is insufficient. It employs albumin dialysis to remove both albumin-bound and water-soluble toxins, such as bilirubin and bile acids, thereby improving hepatic encephalopathy, hemodynamic status, and overall toxin burden while allowing time for hepatic recovery. Although it does not replace the liver’s synthetic or metabolic functions, MARS can serve as a bridge to recovery or transplantation in fulminant hepatic failure. Its use is generally limited to critically ill patients in specialized centers where liver dysfunction progresses despite optimal supportive care [16–18]. As MARS is not available in Albania, our patient was managed exclusively with supportive therapy, focusing on hemodynamic stabilization, correction of metabolic disturbances, and close monitoring of liver and renal function to preserve organ perfusion and facilitate spontaneous recovery. Although MARS represents a dedicated extracorporeal liver support therapy, continuous renal replacement therapy (CRRT) and intermittent hemodialysis are commonly used as supportive alternatives in centers where MARS is unavailable. In Albania, CRRT and intermittent hemodialysis are routinely available and may provide metabolic and renal support in acute liver failure, although they do not fully replicate the albumin-bound toxin clearance achieved by MARS. In our case, however, the patient presented more than 48 h after ingestion, when hepatic injury was already established, limiting the potential benefit of extracorporeal toxin removal therapies. Furthermore, renal impairment and metabolic acidosis were not severe, while hemodynamic status and urinary output remained preserved throughout hospitalization. Patients with acute liver failure require intensive care with continuous monitoring of vital signs, neurological status, and laboratory parameters, along with careful management of fluids, electrolytes, and acid–base balance. Hemodynamic support with fluids and vasopressors, prevention and treatment of cerebral edema, selective correction of coagulopathy, renal support including renal replacement therapy when indicated, infection surveillance with timely treatment, metabolic and nutritional optimization, and respiratory support all constitute essential components of care. The goal of supportive management is to stabilize organ function, prevent complications, and maintain metabolic homeostasis while definitive therapies, including liver transplantation, are evaluated [19]. Table 11 summarizes general supportive treatment strategies [20]. Supportive care in acute liver failure is inherently multisystem, aiming to stabilize hemodynamics,preventcomplications, and preserve organ function while definitive interventions, including transplantation, are considered. Fluid management requires careful assessment and guided resuscitation with balanced crystalloids, avoiding both hypovolemia and overload [20]. Noradrenaline is the vasopressor of choice for fluid-refractory hypotension, maintaining adequate mean arterial pressure and organ perfusion while limiting ischemic risk [21]. Cerebral edema, a major determinant of morbidity, is managed through minimal stimulation, gentle suctioning, strict glucose control, and electrolyte optimization, targeting serum sodium levels of 145–155 mEq/L with hypertonic saline or mannitol when indicated [22, 23]. NAC offers hepatoprotective benefits in both paracetamol and non-paracetamol acute liver failure, improving short-term outcomes. Early initiation of CRRT supports ammonia clearance, corrects metabolic disturbances, and assists in fluid balance [24]. Mild therapeutic hypothermia may reduce cerebral edema and metabolic demand, although supporting evidence remains limited. Mechanical ventilation is reserved for advanced encephalopathy to protect the airway and manage intracranial pressure [25]. Attention to phosphate balance supports neurological recovery, while treatments commonly used for hyperammonemia in chronic liver disease are ineffective in the acute setting [26]. In our patient, hemodynamic stability was achieved through cautious fluid resuscitation with Ringer’s lactate and targeted vasopressor use, in line with recommendations to avoid both hypovolemia and fluid overload. Cerebral edema was managed with close monitoring, glucose control, and electrolyte optimization, while advanced measures such as hypertonic saline or mannitol were reserved for clear indications. NAC was administered despite delayed presentation, reflecting guideline recognition that late treatment may still provide benefit. Additional supportive measures included oxygen therapy, fluid management, temperature control, and targeted correction of coagulopathy and hypoglycemia, consistent with intensive care unit-level monitoring and complication prevention. Limitations were primarily related to resource availability: advanced liver support systems such as MARS and adjunctive antidotes like methionine or fomepizole were not accessible, necessitating reliance on conventional supportive care while arranging transplant evaluation. Overall, management adhered closely to EASL-recommended principles, adapted to local constraints and delayed presentation, ultimately stabilizing multiorgan dysfunction and enabling complete clinical recovery.

**Table 10:** Acetaminophen Liver Damage Stages [5, 6]

Stage	Time frame	Clinical features	Laboratory/organ findings
Stage I	30 min to 24 h	Possibly asymptomatic; nausea, vomiting, diaphoresis, pallor, lethargy, malaise	Transaminases usually normal (may rise after 8–12 h in large ingestions); possible CNS depression and high anion gap metabolic acidosis
Stage II	24–72 h	Apparent clinical improvement; right upper quadrant pain; hepatomegaly	Rising transaminases; worsening labs despite clinical improvement; increased PT and bilirubin; possible renal injury/oliguria; rare acute pancreatitis
Stage III	72–96 h	Severe symptoms: jaundice, encephalopathy, bleeding tendency; recurrence of early symptoms	Peak aminotransferases (> 10,000 IU/L); hyperammonemia; prolonged PT; hypoglycemia; lactic acidosis; indirect hyperbilirubinemia; acute renal failure common; highest mortality risk (multisystem organ failure)
Stage IV	≥ Day 4 (recovery phase)	Clinical recovery if survival of stage III	Gradual normalization of labs (weeks); histopathologic liver changes evident; typically, no long-term liver dysfunction or cirrhosis

CNS: central nervous system; PT: prothrombin time.

**Table 11:** General Supportive Treatment’s Modalities [18]

System/complication	Supportive measures
General/ICU care	Continuous monitoring of vital signs, neurological status, labs; manage fluids and electrolytes; ICU admission required
Hemodynamic	Maintain blood pressure and organ perfusion; use fluids and vasopressors as needed; avoid fluid overload
Cerebral edema/intracranial hypertension	Elevate head of bed, normothermia, sedation; osmotic therapy (mannitol) if ICP rises; avoid hypercapnia and hyponatremia
Coagulation	Correct only if active bleeding or invasive procedures; routine prophylaxis not recommended; use FFP/platelets selectively
Renal	Monitor kidney function; initiate renal replacement therapy for severe metabolic derangements or volume overload
Infection	Frequent microbiological monitoring: prompt antibiotic/antifungal therapy if infection develops
Metabolic/nutrition	Maintain glucose homeostasis; enteral nutrition preferred; parenteral if enteral contraindicated
Respiratory	Mechanical ventilation if severe encephalopathy or respiratory failure develops

FFP: fresh frozen plasma; ICP: intracranial pressure; ICU: intensive care unit.

### Conclusions

Comprehensive supportive care including correction of hepatic, renal, central nervous system, metabolic, and coagulation abnormalities successfully prevented life-threatening complications, halted progression to irreversible organ damage, and provided sufficient time for hepatic recovery, even in the absence of specific antidotal therapy.

### Learning points

Combined overdose with acetaminophen and ibuprofen can lead to severe multi-organ toxicity, particularly when presentation is delayed.

NAC should be administered even in delayed presentations, as it may still provide hepatoprotective effects and improve outcomes.

In resource-limited settings where antidotes and advanced therapies are unavailable, meticulous supportive care becomes the cornerstone of management and may be lifesaving.

Comprehensive supportive management must address hepatic failure, metabolic acidosis, coagulopathy, renal dysfunction, and hypoglycemia, with close monitoring in an intensive care setting.

Advanced extracorporeal liver support systems such as MARS can serve as a bridge to recovery or transplantation but are not universally accessible.

Even in severe acute liver failure with extreme biochemical derangements, favorable outcomes may be possible without liver transplantation when complications are effectively controlled.

This case underscores the critical importance of early recognition, aggressive supportive therapy, and sustained monitoring, especially in delayed presentations and low-resource environments.

## Data Availability

The authors declare that data supporting the findings of this study are available within the article.
